# Comparison of Intestinal Bacteria of *Procambarus clarkii* Farmed in Various Rice Paddy Regions

**DOI:** 10.3390/ani14060935

**Published:** 2024-03-19

**Authors:** Chonghang Ding, Rui Jia, Yunfeng Wang, Yiran Hou, Liqiang Zhang, Bing Li, Jian Zhu

**Affiliations:** 1 College of Fisheries and Life Science, Shanghai Ocean University, Shanghai 201306, China; dingchonghang1999@163.com; 2 Key Laboratory of Integrated Rice-Fish Farming Ecology, Ministry of Agriculture and Rural Affairs, Freshwater Fisheries Research Center, Chinese Academy of Fishery Sciences, Wuxi 214081, China; jiar@ffrc.cn (R.J.); houyr@ffrc.cn (Y.H.); zhangliqiang@ffrc.cn (L.Z.); 3 Fishery Technology Extension Station of Yunnan, No. 25 Dianchi Road, Xishan District, Kunming 650034, China; wangyunfengyunnan@163.com

**Keywords:** *Procambarus clarkii*, integrated rice–crayfish co-culture, intestinal microbiota, microbial diversity, co-occurrence networks

## Abstract

**Simple Summary:**

*Procambarus clarkii*, an important freshwater species in China, is predominantly cultivated in an integrated rice–crayfish co-culture system. However, potential regional differences in the quality of *P. clarkii* have been scarcely reported. Therefore, the aim of this study was to assess the regional differences of *P. clarkii* through analyzing gut microbiota composition in specimens from ten major producing areas in China. Microbial sequencing analysis demonstrated that *P. clarkii* across various regions exhibit distinct microbial diversity and composition. These data would contribute to the assessment of the quality of *P. clarkii* aquaculture.

**Abstract:**

The aim of this study was to assess the regional differences of *Procambarus clarkii* through analyzing gut microbiota in specimens from different areas in China. The *P. clarkii* were collected from ten integrated rice–crayfish farming systems locating across ten major producing areas as follows: Feixi (FX), Suqian (SQ), Yangzhou (YZ), Xuyi (XY), Qianjiang (QJ), Jianli (JL), Honghu (HH), Yueyang (YY), Changsha (CS), and Nanxian (NX). The composition of gut microbiota was assessed by analyzing 16S rRNA sequences. The PCoA results indicated significant differences in microbial community composition among the ten areas (R = 0.999, *p* = 0.001). The intestinal microbial diversity in *P. clarkii* cultured in rice fields from YY and CS exceeded that of other regions, with NX displaying the least diversity. At the phylum level, Proteobacteria were most abundant in HH, while Firmicutes showed increased relative abundances in FX and SQ, contrasted by lower relative abundances of Bacteroidetes in these areas. At the genus level, *Ralstonia*, *Amedibacillus*, *Bacteroides*, *Anaerorhabdus*, and *Dysgonomonas* were the dominant bacteria. The bacterial co-occurrence networks analysis revealed that the community structures in locations FX, SQ, XY, HH, and NX were comparatively simplistic, whereas those in the YZ, QJ, JL, YY, and CS regions displayed as more complex. In summary, the diversity and relative abundance of intestinal bacteria exhibits regional variability. These findings can offer theoretical data for evaluating the quality of *P. clarkii* aquaculture.

## 1. Introduction

The red swamp crayfish (*Procambarus clarkii*), an important freshwater species in China, holds considerable economic value [1]. It is highly valued for artificial breeding due to its rapid growth, substantial fecundity, and broad dietary range [2]. The scale of *P. clarkii* aquaculture has experienced annual growth, reaching a production volume of 2.89 million tons in 2022. The regions leading in *P. clarkii* production include Hubei, Hunan, Jiangsu, and Anhui, situated in eastern and central China [3]. *P. clarkii* cultivation practices encompass various models such as pond monoculture, integrated rice–crayfish farming, and lotus root field culture. Among these models, integrated rice–crayfish co-culture is the predominant cultivation model [4]. The robust expansion of the crayfish industry is largely attributable to the optimization of the integrated rice–crayfish farming approach [5].

Integrated rice–crayfish farming is a sustainable agricultural production system that aims to reduce pollution, enhance ecological stability, and improve economic benefits [6]. By integrating rice and crayfish farming, a mutually beneficial relationship is established between the two. The rice field offers an abundant food source for crayfish, including aquatic insects, zooplankton, and phytoplankton. In turn, crayfish help maintain the health of the paddy field by consuming excess nutrients, thereby preventing the outbreak of pests and pathogens [7]. In this system, crayfish farming can improve soil fertility and increase soil carbon and nitrogen content [8]. It can effectively achieve resource conservation, environment protection, and ecological balance [5]. In 2022, integrated rice–crayfish farming spanned 1.57 million hectares and yielded 2.40 million tons, representing 83% of China’s total cultured crayfish production [9]. The predominant crayfish-producing provinces (Hubei, Jiangsu, Hunan, Anhui, and Jiangxi) exhibited high yields and expansive cultivation territories, collectively comprising over 90% of China’s rice–crayfish farming area and production [10].

Intestinal bacteria in aquatic animals significantly contribute to host metabolism, growth, and immunity, serving as an essential factor for maintaining aquatic animal health [11]. Some microbiotas can produce specific metabolites through their unique metabolic functions, which can significantly affect the growth performance of aquatic animals and accelerate or inhibit the host’s nutrient absorption efficiency and energy acquisition [7]. Its composition and diversity are highly susceptible to the farming environment, such as the water quality and temperature. In addition, age and feed composition can also significantly affect the composition and diversity of intestinal bacteria [12,13]. The relative abundance of intestinal bacteria in *Litopenaeus vannamei* exhibited significant variation between individuals cultured in freshwater and those cultured in marine environments [14]. Furthermore, the distinct changes in flavor observed in *E. sinensis* from different regions can be attributed to variations in gut microbial composition [15]. From summer to autumn, *P. clarkii* cultivated in integrated rice–crayfish farming systems exhibit more stability in an intestinal microbial community than those raised in a pond model [16].

Several comparative studies have been conducted on the integrated rice–crayfish farming in various regions, but most of these studies have primarily focused on changes in the farming environment [17,18] and the accumulation of harmful substances, such as heavy metals [19,20] and microplastics [21]. However, there has been limited research comparing the intestinal microbial composition of *P. clarkii* farmed in various rice paddy regions. Therefore, in this study, we collected *P. clarkii* samples from ten principal production regions in China and analyzed the composition of gut microbiota with 16S rRNA sequencing. These findings may provide a reference for enhancing crayfish farming methods and facilitating quality control.

## 2. Materials and Methods

### 2.1. Sample Collection and Pretreatment

In this study, *P. clarkii* were collected from ten integrated rice–crayfish farming systems. These farms were located across five different provinces: Feixi County (FX) in Hefei, Anhui Province; Suqian city (SQ), Yangzhou city (YZ), and Xuyi (XY) of Huaian city, Jiangsu Province; Qianjiang (QJ), Jianli (JL) and Honghu (HH) all in Jingzhou city, Hubei Province; and Yueyang city (YY), Changsha city (CS), and Nanxian (NX) of Yiyang city, Hunan Province. Intestinal contents were collected from 200 *P. clarkii* per region, with a sex ratio of 1:1, and the contents from 20 individuals were pooled to create a single sample. The collection period was from 20 June to 10 July in 2022, along with a strictly controlled consistency of sampling. The average weight of the *P. clarkii* ranged from 25.18 to 38.61 g. All samples were stored at −80 °C until analysis.

### 2.2. DNA Extraction, PCR Amplification, and Sequencing DNA

Microbial DNA was isolated from the *P. clarkii* intestinal samples employing the E.Z.N.A.^®^ Soil DNA Kit (Omega Bio-tek, Norcross, GA, USA). The integrity and concentration of the extracted DNA were assessed utilizing a NanoDrop 2000 Spectrophotometer (Bio-Rad Laboratories, Inc., Hercules, CA, USA). The polymerase chain reaction (PCR) amplification was performed specifically on the V3-V4 hypervariable regions of the bacterial 16S rRNA gene, utilizing the primers 515F (5′-barcode -GTGCCAGCMGCCGCGG-3′) and 907R (5′-CCGTCAATTCMTTTRAGTTT-3′). The PCR products were purified with the AxyPrep DNA Gel Extraction Kit (Axygen Biosciences, Union City, CA, USA) and quantified using the QuantiFluor™-ST (Promega, Madison, WI, USA).

### 2.3. Statistical Analysis

All data were analyzed using SPSS software (version 25.0). A one-way analysis of variance (ANOVA) with post hoc LSD was used to compare the significant differences between the different groups. The values were expressed as mean ± SE (standard error). Statistical significance was set at *p* < 0.05. The results were plotted using GraphPad Prism 8.0 software.

Purified PCR products were used to construct a DNA library, which was sequenced on an Illumina MiSeq platform (Shanghai BIOZERON Co., Ltd., Shanghai, China). The raw data were processed using the DADA2 R package (version 1.14) to eliminate low-quality sequences and chimeras, then to produce amplicon sequence variants (ASVs). Sequence alignment was performed using the UCLUST (v1.2.22q) based on the SILVA database. The QIIME software (version 1.9.1) were utilized to compute Alpha diversity metrics, including Chao1, Shannon, Simpson, and evenness. For the visualization of community composition relationships, principal coordinate analysis (PCoA) predicated on weighted UniFrac distances was enacted using R project. Additionally, the analysis of similarities (ANOSIM) was performed to evaluate the statistical significance between different groups, employing the Vegan package (version 2.5.3).

## 3. Results

### 3.1. Intestinal Microbiota Alpha-Diversity Analysis

**Figure 1:**
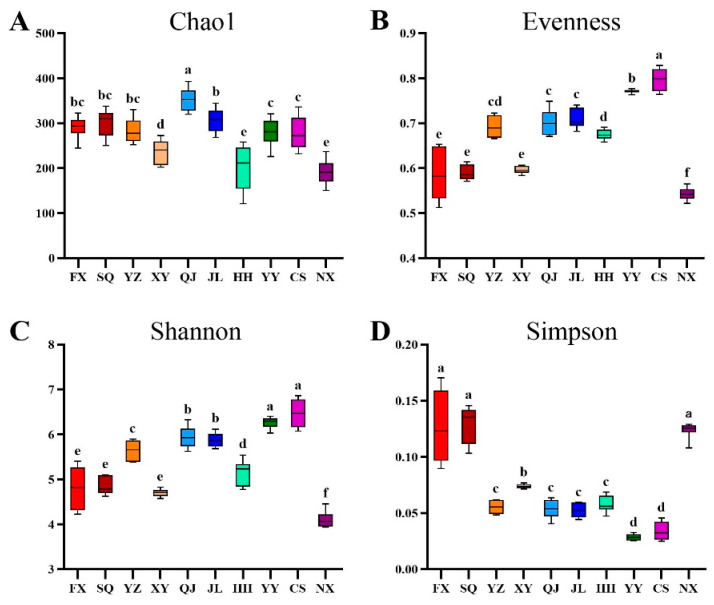
The alpha diversity analysis for intestinal bacteria of *P. clarkii* from different areas: (**A**) Chao1 index; (**B**) evenness; (**C**) Shannon index and (**D**) Simpson index. The different letters indicate significant difference (*p* < 0.05). FX, Feixi County; SQ, Suqian city; YZ, Yangzhou city; XY, Xuyi County; QJ, Qianjiang city; JL, Jianli County; HH, Honghu city; YY, Yueyang city; CS, Changsha city; NX, Nanxian County.

The gut microbes of *P. clarkii* were compared in three dimensions of richness, evenness, and diversity, in which the Chao1 index was used to indicate richness, and Shannon and Simpson indices were used to quantify diversity (Table S2). The results are presented in Figure 1, indicating that QJ and JL significantly exhibited a higher species richness (*p* < 0.05). In contrast, NX, XY, and HH displayed a significantly lower richness (*p* < 0.05). As for species evenness, CS and YY ranked the highest, while NX demonstrated the least evenness, with FX, SQ, and XY also showing notably lower values, exhibiting significant differences (*p* < 0.05). CS and YY exhibited a significantly higher diversity compared to other areas, while NX, FX, and SQ had a significantly lower diversity (*p* < 0.05). Overall, the *P. clarkii* gut bacterial communities in YY and CS demonstrated higher richness and diversity compared to those in other regions. Conversely, these attributes were markedly lower in NX, with SQ, FX, and XY also showing reduced levels.

### 3.2. Intestinal Microbiota Beta-Diversity Analysis

**Figure 2:**
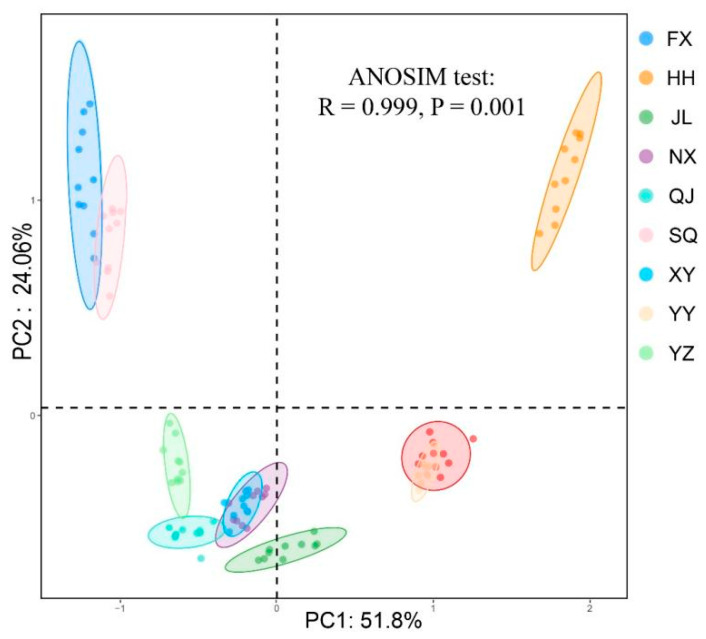
Principal coordinate analysis (PCoA) of gut microbial communities based on weighted unifrac. FX, Feixi County; SQ, Suqian city; YZ, Yangzhou city; XY, Xuyi County; QJ, Qianjiang city; JL, Jianli County; HH, Honghu city; YY, Yueyang city; NX, Nanxian County.

**Figure 3:**
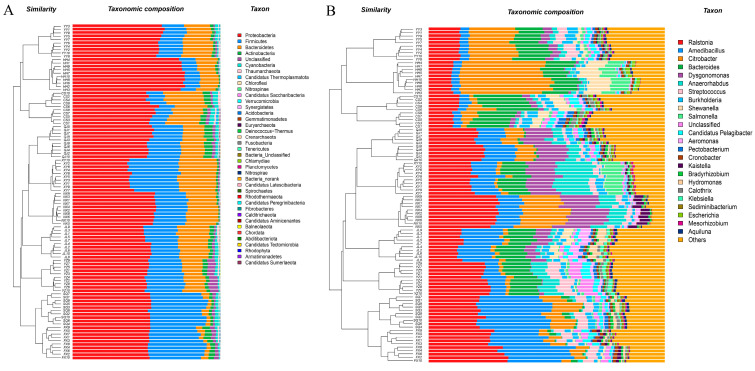
Cluster analysis (Bray–Curtis) of intestinal bacteria at the phylum (**A**) and genus (**B**) levels.

To assess the variability of gut microbiota across different regions, a principal coordinate analysis (PCoA) was performed, which yielded PC1 explaining 51.80% of the variation and PC2 accounting for 24.06%. The results of the PCoA revealed distinct clustering patterns among samples from various groups (Figure 2). Additionally, an analysis of similarity (ANOSIM) indicated significant differences in microbial community composition between groups (R = 0.999, *p* = 0.001). Furthermore, cluster analysis (Bray–Curtis) on the phylum and genus levels showed that samples from the same region had a tendency to group together (Figure 3).

### 3.3. Microbial Community Composition

**Figure 4:**
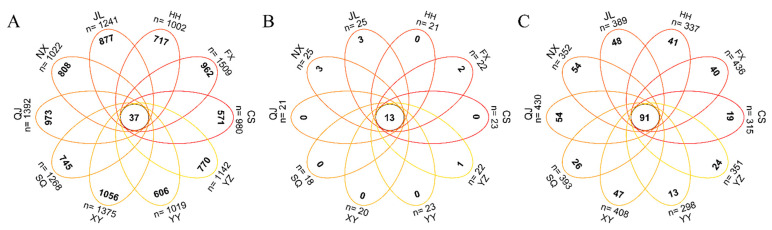
Venn diagram in ASVs (**A**), phylum (**B**), and genus (**C**) of intestinal microbiota in *P. clarkii* from different areas. FX, Feixi County; SQ, Suqian city; YZ, Yangzhou city; XY, Xuyi County; QJ, Qianjiang city; JL, Jianli County; HH, Honghu city; YY, Yueyang city; CS, Changsha city; NX, Nanxian County.

**Figure 5:**
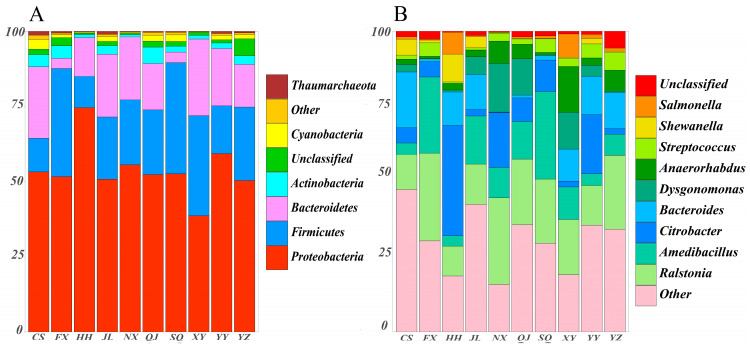
The proportions of intestinal microbiota at phylum level (**A**) and genus level (**B**). FX, Feixi County; SQ, Suqian city; YZ, Yangzhou city; XY, Xuyi County; QJ, Qianjiang city; JL, Jianli County; HH, Honghu city; YY, Yueyang city; CS, Changsha city; NX, Nanxian County.

**Figure 6:**
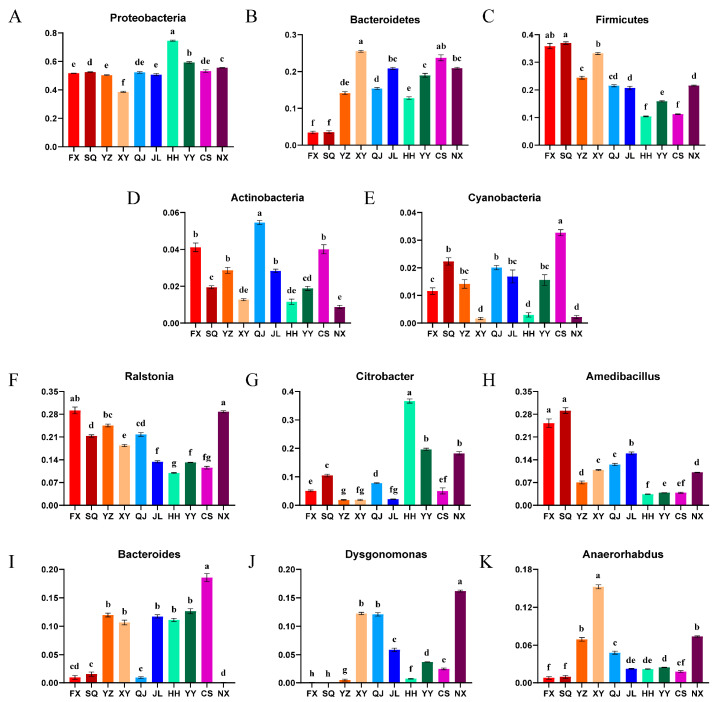
Difference in dominant intestinal microbiota at phylum (**A**–**E**) and genus (**F**–**K**) level. The different letters indicate significant difference (*p* < 0.05). FX, Feixi County; SQ, Suqian city; YZ, Yangzhou city; XY, Xuyi County; QJ, Qianjiang city; JL, Jianli County; HH, Honghu city; YY, Yueyang city; CS, Changsha city; NX, Nanxian County.

Raw data sequences (34,337–43,694) were filtered to produce clean data sequences (33,004–41,997) (Table S1). A total of 9360 ASVs were identified, with 37 ASVs shared across all groups (Figure 4A). At the phylum level, 36 phyla were identified, 13 of which were common across all groups (Figure 4B). At the genus level, 971 genera were identified, with 91 genera shared among the groups (Figure 4C). Microbial compositional analysis indicated that Proteobacteria, Firmicutes, Bacteroidetes, Actinobacteria, and Cyanobacteria were the five most prevalent phyla. Notably, Proteobacteria were the dominant gut bacterial phylum, with its relative abundance exceeding 50% in all regions (except XY, 38.67%). The relative abundance of HH and YY in Proteobacteria was significantly higher than other areas, while XY was the lowest (*p* < 0.05). Bacteroidetes dominated from the gut of *P. clarkii* in XY and CS, but in FX and SQ, were significantly lower. Firmicutes dominated in FX and SQ, but was significantly lower in HH and CS. Meanwhile, the relative abundance of Actinobacteria in QJ and Cyanobacteria in CS was significantly higher than that in other regions (Figure 5A and Figure 6).

A genus-level analysis revealed that there were significant differences in dominant genera in different areas. *Ralstonia* was the dominant genus (>10%, except HH) in all areas, and FX and NX also exhibited significantly higher relative abundances of this genus. Moreover, *Amedibacillus* was the dominant genus in the FX, SQ, and JL regions, while *Bacteroides* prevailed in CS and *Citrobacter* in HH, YY, and NX. *Dysgonomonas* was the leading genus in NX, XY, and QJ, and *Anaerorhabdus* in XY, YZ, and NX regions. The relative abundance of these dominant bacterial genera was significantly higher than in other areas (Figure 5B and Figure 6).

### 3.4. Co-Occurrence Networks of P. clarkii Intestinal Bacterial Communities in Different Areas

**Figure 7:**
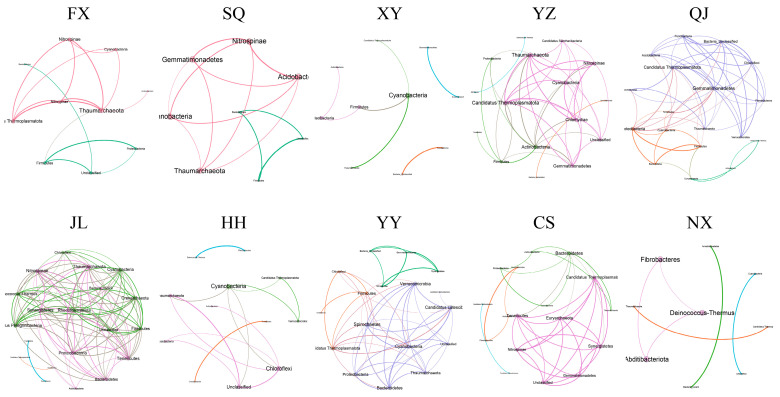
Co-occurrence networks of *P. clarkii* intestinal bacterial communities in different areas. FX, Feixi County; SQ, Suqian city; YZ, Yangzhou city; XY, Xuyi County; QJ, Qianjiang city; JL, Jianli County; HH, Honghu city; YY, Yueyang city; CS, Changsha city; NX, Nanxian County.

An analysis of bacterial co-occurrence networks revealed significant variability in community structure across the different regions (Figure 7). In locations FX, SQ, XY, HH, and NX, the structures were comparatively simplistic. Networks were characterized as follows: FX comprised 10 nodes and 15 edges; SQ encompassed 8 nodes with 13 edges; XY consisted of 10 nodes connected by 7 edges; HH displayed 12 nodes and 14 edges; and NX contained 9 nodes with 6 edges. In contrast, the community structures within the YZ, QJ, JL, YY, and CS regions were more complex. The respective co-occurrence networks for these groups consisted of 16 nodes with 44 edges in YZ, 18 nodes with 57 edges in QJ, 20 nodes with 105 edges in JL, 17 nodes with 51 edges in YY, and 17 nodes with 36 edges in CS. (Table 1). In addition, we compared the ratio of positive to negative edges across various regional networks. The results indicated that the ratio of positive edges exceeded 50% in all examined regional networks, with particularly high ratios observed in FX, XY, HH, and NX, reaching levels as elevated as 70% and, in some cases, up to 100%.

## 4. Discussion

Gut microbes compose a complex and dynamic microbial community [19] that influences host growth, metabolism, immunity, and overall health [16,22]. This microbiome is subject to various factors in the aquaculture, including the growth stage of the host, feed composition, and an array of environmental conditions [23,24,25]. The diversity of the gut microbiota is widely recognized as an indicator of host health [26]. Numerous studies have highlighted the close connection between the gut microbiota of aquatic animals and that of their environmental counterparts [27,28]. In this experiment, β-diversity revealed distinct clustering by region, suggesting that varying farming environments markedly influence microbial community composition. A higher α-diversity in the intestinal microbiome was indicative of stability, which in turn was associated with a beneficial impact on crayfish health. The intestinal bacterial communities in YY and CS *P. clarkii* exhibited superior structure, abundance, and diversity compared to those in other regions, which may denote a more stable and growth-favorable intestinal microbiome. Conversely, NX displayed the least-structured and diverse microbial community, paralleled by SQ, FX, and XY, potentially increasing susceptibility to diseases in *P. clarkii*.

Our results also showed that the predominant bacterial phyla in *P. clarkii* include Proteobacteria, Firmicutes, Bacteroidetes, Actinobacteria, and Cyanobacteria, with Proteobacteria being the most prevalent, which was consistent with the bacterial profiles observed in studies of *Procambarus clarkii* [5] and *Litopenaeus vannamei* [29]. Proteobacteria are highly abundant in freshwater ecosystems, where they play a crucial role in the degradation of organic macromolecules [30]. Moreover, these bacteria are commonly found in the gut microbiomes of aquatic invertebrates [31] and represent the predominant microbial phylum in the shrimp gut [32]. Previous studies have demonstrated a higher abundance of Proteobacteria in *P. clarkii* ponds compared to paddy fields [16]. Additionally, the abundance of Proteobacteria of *P. clarkii* from the Hubei province surpasses that of Hunan or Guangdong [10]. However, in this study, Proteobacteria were observed to be the most relevantly abundant across all sampled areas, exhibiting a stable presence with a small relative abundance gap in most areas. This finding suggests that Proteobacteria are a consistent and integral component of *P. clarkii* intestinal microbiota.

Apart from Proteobacteria, Firmicutes and Bacteroidetes represent important phyla in intestinal microbiome of the *P. clarkii*. Firmicutes have been linked to the transformation of complex organic matter such as protein and fat during the growth of the host, providing essential nutrients and energy for the organism [33]. Bacteroidetes aid in the breakdown of carbohydrates and maintaining gut homeostasis [34]. Previous studies have revealed that the abundance of Firmicutes in the gut of *P. clarkii* was higher during summer months compared to autumn, and it was also higher in *P. clarkii* guts cultured in rice fields than in ponds [16]. In this study, we found that the gut microbiota in FX and SQ exhibited a higher relative abundance of Firmicutes, while CS was lower, which may suggest an increased capacity for nutrient transport and energy utilization in *P. clarkii* from FX and SQ. Conversely, the relative abundance of Bacteroidetes in the above areas displayed an inverse trend, which may reflect a potentially antagonistic functional role between these bacterial phyla.

At the genus level, the predominant bacteria identified were *Ralstonia*, *Amedibacillus*, *Bacteroides*, *Citrobacter*, *Anaerorhabdus* and *Dysgonomonas*, with *Ralstonia* being the most prevalent in the intestines of *P. clarkii*. *Ralstonia*, belonging to Proteobacteria, is a core microbe in aquatic animals with a high enzyme-producing capacity [35] and has been considered as a potential probiotic in aquaculture [36]. Studies have found that the abundance of *Ralstonia* in the gut microbes of diseased fish and shrimp was significantly lower than that in healthy individuals [37,38], indicating that *Ralstonia* may be one of the health indicators in cultured organisms. In this study, the relative abundance of *Ralstonia* was found to be higher in all areas, indicative of a good health status of *P. clarkii* across each culture areas. Significantly higher relative abundances in FX and NX, contrasted with lower levels in HH and CS, suggest that the former groups of *P. clarkii* were in better health, potentially as a result of superior farming conditions. *Bacteroides*, a genus of anaerobic bacteria prevalent in both the environment and within aquatic animals [35], possesses the ability to hydrolyze proteins, lipids, cellulose, and other organic compounds [39]. These bacteria confer benefits to the immune response and the maintenance of intestinal homeostasis, thereby exerting a direct or indirect influence on host health [40]. This study observed higher relative abundances of *Bacteroides* in CS and YY, with lower levels detected in NX. It can be inferred that the culture environments of CS and YY may be favorable for promoting organic matter hydrolysis, thus improving metabolic functions and potentially augmenting antibacterial or anti-inflammatory responses, as well as enhancing the immunity of *P. clarkii* [41], but the specific impact still needs further study.

The composition and structure of intestinal microbiota in aquatic organisms are intricate, with most bacteria engaging in interactions that form complex networks. These interactions not only complicate the functions but also enhance the connectivity and stability of the microbial structure [42]. Bacterial co-occurrence networks have been used to assess bacterial community structures and patterns of interconnectedness [43]. Healthy communities are characterized by high levels of interconnectivity, modularity, and dynamism, which contribute to systemic stability and organism health [44,45]. Co-occurrence networks exhibit variability in relation to farming activities [46]. In this study, distinct co-occurrence networks of microbiota were observed across various regions, suggesting that the cultural environment exerts an influence on their composition. The co-occurrence networks in YZ, QJ, JL, YY, and CS were more complex, with more nodes and edges, indicating that the gut microbes in these areas had closer interactions and connections, higher stability, and stronger resistance to interference [47,48]. In addition, we found that the positive edge ratios were more than 50% in all areas, with particularly higher ratios in areas with a simple co-network structure. Positive interactions promote bacterial evolution by improving fitness, suggesting lower competition and niche differentiation among gut microbes [46].

## 5. Conclusions

This study compared the composition and diversity of gut microbiota in *P. clarkii* among various regions. The results revealed significant regional disparities in the levels of diversity and relative abundance of intestinal microbes. The gut microbial diversity and relative abundance in YY and CS surpassed those of other regions, with NX displaying the least. Moreover, the co-occurrence networks analysis showed that the gut microbiota structure might be more stable in YZ, QJ, JL, YY, and CS. These findings can offer theoretical data for evaluating the quality of *P. clarkii* aquaculture.

## Figures and Tables

**Table 1:** Topological parameters of co-occurrence networks based on gut bacterial communities in different areas.

	FX	SQ	XY	YZ	QJ	JL	HH	YY	CS	NX
Nodes	10	8	10	16	18	20	12	17	17	9
Edges	15	13	7	44	57	105	14	51	36	6
Positive edge ratio	80.00%	53.85%	71.43%	52.27%	59.65%	46.67%	100%	49.02%	66.67%	100%
Negative edge ratio	20.00%	46.15%	28.57%	47.73%	40.35%	53.33%	0%	50.98%	33.33%	0%

## Data Availability

The raw data of 16S rRNA used in this study have been submitted to open database NCBI Sequence Read Archive (SRA, PRJNA1055421). All other data are contained within the main manuscript and Supplementary Material.

## Supplementary Materials

The following supporting information can be downloaded at: https://www.mdpi.com/article/10.3390/ani14060935/s1, Table S1: Quality control for the sequencing; Table S2: One-way ANOVA for microbial diversity.

## References

[1] Bi K., Gu W., Wang W. (2008). Sensitive and rapid detection of freshwater crustacean Spiroplasmas by ISRs-sequence-targeted species-specific primers. Eur. Food Res. Technol..

[2] Li Y., Guo X., Cao X., Deng W., Luo W., Wang W. (2012). Population genetic structure and post-establishment dispersal patterns of the red swamp crayfish *Procambarus clarkii* in China. PLoS ONE.

[3] Yu X., Hao X., Dang Z., Yang L. (2023). China crayfish industry development report. China Fish..

[4] Zheng D., Peng X., Zhang X., Zhang J., Zhou Y., Peng L., Xia Z. (2023). Comparative Analysis on Muscle Quality of *Procambarus clarkii* Under Different Aquaculture Models in Hubei Province. Sci. Technol. Food Ind..

[5] Shui Y., Guan Z.B., Liu G.F., Fan L.M. (2020). Gut microbiota of red swamp crayfish *Procambarus clarkii* in integrated crayfish-rice cultivation model. AMB Express.

[6] Jiang Y., Cao C. (2021). Crayfish–rice integrated system of production: An agriculture success story in China. A review. Agron. Sustain. Dev..

[7] Nie Z., Xu X., Shao N., He J., Li P., Xu P., Hu J., Qin W., Wang B., Xu G. (2023). Integrative analysis of microbiome and metabolome reveals the linkage between gut microbiota and carp growth. Environ. Res..

[8] Si G., Peng C., Xu X., Xu D., Yuan J., Li J. (2017). Effect of integrated rice-crayfish farming system on soil physico-chemical properties in waterlogged paddy soils. Chin. J. Eco-Agric..

[9] Yu X., Hao X., Dang Z., Yang L. (2023). Report on the Development of China’s Rice-Fishery Comprehensive Breeding Industry (2023). China Fish..

[10] Jia L., Wang G., Xia Y., Zhang K., Gao S., Li Y., Gao Y. (2022). Comparison of physiological metabolism, muscle quality and nutritional value of *Procambarus clarkii* cultivated in paddy fields in different areas. J. Gansu Agric. Univ..

[11] Sun Y., Han W., Liu J., Huang X., Zhou W., Zhang J., Cheng Y. (2020). Bacterial community compositions of crab intestine, surrounding water, and sediment in two different feeding modes of Eriocheir sinensis. Aquac. Rep..

[12] Huang Z., Li X., Wang L., Shao Z. (2016). Changes in the intestinal bacterial community during the growth of white shrimp, *Litopenaeus vannamei*. Aquac. Res..

[13] Zheng Y., Yu M., Liu J., Qiao Y., Wang L., Li Z., Zhang X.-H., Yu M. (2017). Bacterial Community Associated with Healthy and Diseased Pacific White Shrimp (*Litopenaeus vannamei*) Larvae and Rearing Water across Different Growth Stages. Front. Microbiol..

[14] Fan L., Wang Z., Chen M., Qu Y., Li J., Zhou A., Xie S., Zeng F., Zou J. (2019). Microbiota comparison of Pacific white shrimp intestine and sediment at freshwater and marine cultured environment. Sci. Total Environ..

[15] Tao H., Du B., Wang H., Dong H., Yu D., Ren L., Sima Y., Xu S. (2018). Intestinal microbiome affects the distinctive flavor of Chinese mitten crabs in commercial farms. Aquaculture.

[16] Liu Q., Long Y., Li B., Zhao L., Luo J., Xu L., Luo W., Du Z., Zhou J., Yang S. (2020). Rice-shrimp culture: A better intestinal microbiota, immune enzymatic activities, and muscle relish of crayfish (*Procambarus clarkii*) in Sichuan Province. Appl. Microbiol. Biotechnol..

[17] Yuan P., Wang J., Li C., Xiao Q., Liu Q., Sun Z., Wang J., Cao C. (2020). Soil quality indicators of integrated rice-crayfish farming in the Jianghan Plain, China using a minimum data set. Soil Tillage Res..

[18] Si G., Peng C., Yuan J., Xu X., Zhao S., Xu D., Wu J. (2017). Changes in soil microbial community composition and organic carbon fractions in an integrated rice-crayfish farming system in subtropical China. Sci. Rep..

[19] Zhou H., Ge T., Li H., Fang T., Li H., Shi Y., Zhang R., Dong X. (2022). A Multi-Medium Analysis of Human Health Risk of Toxic Elements in Rice-Crayfish System: A Case Study from Middle Reach of Yangtze River, China. Foods.

[20] Peng F., Li J., Gong Z., Yue B., Wang X., Manyande A., Du H. (2022). Investigation of Bioaccumulation and Human Health Risk Assessment of Heavy Metals in Crayfish (*Procambarus clarkii*) Farming with a Rice-Crayfish-Based Coculture Breeding Modes. Foods.

[21] Zhang D., Fraser M.A., Huang W., Ge C., Wang Y., Zhang C., Guo P. (2021). Microplastic pollution in water, sediment, and specific tissues of crayfish (*Procambarus clarkii*) within two different breeding modes in Jianli, Hubei province, China. Environ. Pollut.

[22] Valdes A.M., Walter J., Segal E. (2018). Role of the gut microbiota in nutrition and health. BMJ.

[23] Cornejo-Granados F., Gallardo-Becerra L., Leonardo-Reza M., Ochoa-Romo J.P., Ochoa-Leyva A. (2018). A meta-analysis reveals the environmental and host factors shaping the structure and function of the shrimp microbiota. PeerJ.

[24] Li X., Li S., Shi G., Luo X., Kang J., Su J., Wang L. (2021). Effects of Growth Environment on Bacterial Community Diiversity of *Procambarus clarkia*. Sci. Technol. Food Ind..

[25] Li X., Zhou L., Yu Y., Ni J., Xu W., Yan Q. (2017). Composition of Gut Microbiota in the Gibel Carp (*Carassius auratus* gibelio) Varies with Host Development. Microb. Ecol..

[26] Ren Z., Li A., Jiang J., Zhou L., Yu Z., Lu H., Xie H., Chen X., Shao L., Zhang R. (2019). Gut microbiome analysis as a tool towards targeted non-invasive biomarkers for early hepatocellular carcinoma. Gut.

[27] Hou D., Huang Z., Zeng S., Liu J., Weng S., He J. (2018). Comparative analysis of the bacterial community compositions of the shrimp intestine, surrounding water and sediment. J. Appl. Microbiol..

[28] Xiong J., Dai W., Qiu Q., Zhu J., Yang W., Li C. (2018). Response of host-bacterial colonization in shrimp to developmental stage, environment and disease. Mol. Ecol..

[29] Fan J., Chen L., Mai G., Zhang H., Yang J., Deng D., Ma Y. (2019). Dynamics of the gut microbiota in developmental stages of *Litopenaeus vannamei* reveal its association with body weight. Sci. Rep..

[30] Eiler A., Bertilsson S. (2004). Composition of freshwater bacterial communities associated with cyanobacterial blooms in four Swedish lakes. Environ. Microbiol..

[31] Holt C.C., Bass D., Stentiford G.D., van der Giezen M. (2021). Understanding the role of the shrimp gut microbiome in health and disease. J. Invertebr. Pathol..

[32] Xiong J., Zhu J., Dai W., Dong C., Qiu Q., Li C. (2017). Integrating gut microbiota immaturity and disease-discriminatory taxa to diagnose the initiation and severity of shrimp disease. Environ. Microbiol..

[33] Shi J., Wu X., Huang G., Yang H., Lliang Y., Lyu M., Zeng L., Hu D., Huang L., Wang R. (2023). Structural and Functional Characteristics of Intestinal Microbiota in Different Populations of *Macrobrachium rosenbergii* Larvae. Fish. Sci..

[34] Li M., Shang Q., Li G., Wang X., Yu G. (2017). Degradation of Marine Algae-Derived Carbohydrates by Bacteroidetes Isolated from Human Gut Microbiota. Mar. Drugs.

[35] Ramirez R.F., Dixon B.A. (2003). Enzyme production by obligate intestinal anaerobic bacteria isolated from oscars (*Astronotus ocellatus*), angelfish (*Pterophyllum scalare*) and southern flounder (*Paralichthys lethostigma*). Aquaculture.

[36] Van Hung N., De Schryver P., Dung N.V., Nevejan N., Bossier P. (2019). *Ralstonia eutropha*, containing high poly-beta-hydroxybutyrate levels, regulates the immune response in mussel larvae challenged with *Vibrio coralliilyticus*. Fish Shellfish Immunol..

[37] Leng X., Luo J., Du H., Xiong W., Qiao X., Wei Q. (2021). Alterations in the gut microbiota in Chinese sturgeon (*Acipenser sinensis*) suffering from haemorrhagic septicaemia. Aquac. Res..

[38] Yu P., Shan H., Cheng Y., Ma J., Wang K., Li H. (2022). Translucent disease outbreak in *Penaeus vannamei* post-larva accompanies the imbalance of pond water and shrimp gut microbiota homeostasis. Aquac. Rep..

[39] Zhang X., Tao Y., Hu J., Liu G., Spanjers H., van Lier J.B. (2016). Biomethanation and microbial community changes in a digester treating sludge from a brackish aquaculture recirculation system. Bioresour. Technol..

[40] Yang E.-J., Amenyogbe E., Li R.-X., Zhang J.-D., Xie R.-T., Wang Z.-L., Chen G., Huang J.-S., Kumar P. (2023). The Microflora Structure in the Digestive Tract, Culture Water, and Feed of Hybrid Grouper (*Epinephelus fuscoguttatus*♀ × *E. polyphekadion*♂) Cultured in an Outdoor Pond Based on a High-Throughput Sequencing Technique. Aquac. Res..

[41] Chapagain P., Walker D., Leeds T., Cleveland B.M., Salem M. (2020). Distinct microbial assemblages associated with genetic selection for high- and low- muscle yield in rainbow trout. BMC Genom..

[42] Zhao Y., Guo H., Zhang D. (2021). Effects of different culture patterns on the intestinal microbiota of *Litopenaeus vannamei*. J. Fish. China.

[43] Hou Y., Li B., Xu G., Li D., Zhang C., Jia R., Li Q., Zhu J. (2021). Dynamic and Assembly of Benthic Bacterial Community in an Industrial-Scale In-Pond Raceway Recirculating Culture System. Front. Microbiol..

[44] Lu L., Yin S., Liu X., Zhang W., Gu T., Shen Q., Qiu H. (2013). Fungal networks in yield-invigorating and -debilitating soils induced by prolonged potato monoculture. Soil Biol. Biochem..

[45] Tseng D.Y., Ho P.L., Huang S.Y., Cheng S.C., Shiu Y.L., Chiu C.S., Liu C.H. (2009). Enhancement of immunity and disease resistance in the white shrimp, *Litopenaeus vannamei*, by the probiotic, *Bacillus subtilis* E20. Fish Shellfish Immunol..

[46] Zhou M., Hou Y., Jia R., Li B., Zhu J. (2023). Effects of *Bellamya purificata* Cultivation at Different Stocking Densities on the Dynamics and Assembly of Bacterial Communities in Sediment. Biomolecules.

[47] de Vries F.T., Griffiths R.I., Bailey M., Craig H., Girlanda M., Gweon H.S., Hallin S., Kaisermann A., Keith A.M., Kretzschmar M. (2018). Soil bacterial networks are less stable under drought than fungal networks. Nat. Commun..

[48] Fisher R.M., Henry L.M., Cornwallis C.K., Kiers E.T., West S.A. (2017). The evolution of host-symbiont dependence. Nat. Commun..

