# Supplementary material for: Comparison of Intestinal Bacteria of Procambarus clarkii Farmed in Various Rice Paddy Regions
Source: Animals (Basel). 2024 Mar 19;14(6):935. doi: 10.3390/ani14060935 (PMC10967483; doi:10.3390/ani14060935)
Supplement: Supplementary file 1 [file animals-14-00935-s001.zip › animals-2911419-supplementary.pdf]

Table S1. Quality control for the sequencing.

Sample	Raw data	Clean data	Sample	Raw data	Clean data
FX1	42299	40656	XY1	37732	36267
FX2	41326	39721	XY2	36438	35023
FX3	38483	36989	XY3	40078	38522
FX4	40587	39011	XY4	34398	33062
FX5	37709	36245	XY5	41689	40070
FX6	40570	38995	XY6	43265	41585
FX7	41529	39916	XY7	38948	37436
FX8	43110	41436	XY8	43109	41435
FX9	41804	40181	XY9	38337	36848
FX10	43182	41505	XY10	41136	39539
SQ1	42593	40939	CS1	34793	33442
SQ2	36948	35513	CS2	42461	40812
SQ3	42070	40436	CS3	37094	35654
SQ4	39631	38092	CS4	34347	33013
SQ5	38400	36909	CS5	43239	41560
SQ6	40549	38974	CS6	36336	34925
SQ7	43225	41547	CS7	38257	36771
SQ8	38730	37226	CS8	41118	39521
SQ9	42869	41204	CS9	37109	35668
SQ10	36691	35266	CS10	38787	37281
YZ1	36958	35523	HH1	36598	35177
YZ2	40366	38799	HH2	41437	39828
YZ3	39856	38308	HH3	37025	35587
YZ4	38521	37025	HH4	34647	33302
YZ5	43440	41753	HH5	42231	40591
YZ6	41133	39536	HH6	42757	41097
YZ7	43428	41742	HH7	37647	36185
YZ8	42193	40555	HH8	35724	34337
YZ9	38213	36729	HH9	39742	38199
YZ10	38275	36789	HH10	43394	41709
JL1	43043	41372	YY1	43189	41512
JL2	43126	41451	YY2	34760	33410
JL3	39158	37637	YY3	39558	38022
JL4	40872	39285	YY4	35881	34488
JL5	42901	41235	YY5	40215	38653
JL6	37049	35610	YY6	38017	36541
JL7	36734	35308	YY7	36447	35032
JL8	42107	40472	YY8	40770	39187
JL9	43639	41944	YY9	35530	34150
JL10	34337	33004	YY10	41313	39709
QJ1	42449	40801	NX1	40988	39396

QJ2	36908	35475	NX2	39913	38363
QJ3	34910	33554	NX3	35233	33865
QJ4	34397	33061	NX4	35445	34069
QJ5	37404	35952	NX5	35049	33688
QJ6	34618	33274	NX6	37965	36491
QJ7	36255	34847	NX7	36287	34878
QJ8	36825	35395	NX8	43694	41997
QJ9	38352	36863	NX9	36625	35203
QJ10	39444	37912	NX10	40142	38583

Table S2. One-way ANOVA for microbial diversity.

Diversity index		Sum of Squares	df	Mean Square	F	Sig.
Chao1	Between Groups	217927.69	9	24214.188	27.499	＜0.001
	Within Groups	79250.1	90	880.557		
	Total	297177.79	99			
Evenness	Between Groups	0.642	9	0.071	117.885	＜0.001
	Within Groups	0.054	90	0.001		
	Total	0.697	99			
Shannon	Between Groups	53.204	9	5.912	97.234	＜0.001
	Within Groups	5.472	90	0.061		
	Total	58.676	99			
Simpson	Between Groups	0.133	9	0.015	100.483	＜0.001
	Within Groups	0.013	90	0		
	Total	0.147	99			
